# Role of RAS signaling in ovarian cancer

**DOI:** 10.12688/f1000research.126337.1

**Published:** 2022-11-04

**Authors:** Lubna Therachiyil, Anjana Anand, Abdullah Azmi, Ajaz Bhat, Hesham M. Korashy, Shahab Uddin

**Affiliations:** 1 Hamad Medical Corporation, Doha, Qatar, 3050, Qatar; 2 Department of Pharmaceutical Sciences, College of Pharmacy, QU Health, Qatar University, Doha, 2713, Qatar; 3 Sidra Medicine, Doha, 26999, Qatar

**Keywords:** Ovarian cancers, RAS, Oncogene, mutation, cell signaling

## Abstract

The RAS family of proteins is among the most frequently mutated genes in human malignancies. In ovarian cancer (OC), the most lethal gynecological malignancy, *RAS*, especially *KRAS* mutational status at codons 12, 13, and 61, ranges from 6–65% spanning different histo-types. Normally RAS regulates several signaling pathways involved in a myriad of cellular signaling cascades mediating numerous cellular processes like cell proliferation, differentiation, invasion, and death. Aberrant activation of RAS leads to uncontrolled induction of several downstream signaling pathways such as RAF-1/MAPK (mitogen-activated protein kinase), PI3K phosphoinositide-3 kinase (PI3K)/AKT, RalGEFs, Rac/Rho, BRAF (v-Raf murine sarcoma viral oncogene homolog B), MEK1 (mitogen-activated protein kinase kinase 1), ERK (extracellular signal-regulated kinase), PKB (protein kinase B) and PKC (protein kinase C) involved in cell proliferation as well as maintenance pathways thereby driving tumorigenesis and cancer cell propagation. *KRAS* mutation is also known to be a biomarker for poor outcome and chemoresistance in OC. As a malignancy with several histotypes showing varying histopathological characteristics, we focus on reviewing recent literature showcasing the involvement of oncogenic *RAS* in mediating carcinogenesis and chemoresistance in OC and its subtypes.

## Introduction

Gynecological malignancies in women’s reproductive organs seriously threaten female lives. Primarily classified based on the organ affected, gynecological cancers are of five major types, ovarian, cervical, uterine, vaginal, and vulvar. ^1^^,^^2^ OC is the most lethal gynecological malignancy and the fifth prominent cause of death in females worldwide. ^3^ Characterized by the poor outcome and relatively lower survival rate, OC is presented with several gene mutations. ^4^ Until now, four major gene mutations are stated to have highly correlated to the occurrence of OC including *TP53, KRAS, BRCA1/2* and *PIK3CA*, ultimately leading to several characteristics of the tumor cells, including abnormal DNA repair mechanisms, impaired tumor suppression, oncogene gain of function, and epigenetic inactivation. ^4^^,^^5^ In OC, *KRAS* mutation is one of the most frequently observed abnormalities. ^6^ Though typically considered to be a single disease, OC is classified into various sub types based on the origin of the tumor and the cellular histology. ^7^

RAS is a family of intrinsic GTP-binding proteins involved in various crucial cellular signal transduction pathways that fundamentally regulate cell growth, differentiation, cell adhesion and migration, and survival. ^8^^–^^10^ Among the small G-proteins, the RAS subfamily is the most studied, due to their crucial involvement in human tumorigenesis. ^11^ RAS is one of the major pathways found to be the most frequently mutated in several cancers, including pancreatic, ^12^ lung, ^13^ colorectal, ^14^^,^^15^ ovarian, ^16^ and hematopoietic malignancies. ^17^^,^^18^

Even though OC is majorly driven by several genetic mutations, ^19^ the role and involvement of *RAS* mutation in this cancer have been scarcely reviewed before. In this review, we will discuss the significance of RAS, its mutational status, and its role in the pathogenesis of different histological types of OC.

### RAS signaling pathway

The RAS superfamily comprises more than 170 members, ^20^ which can be classified into five major protein subfamilies: RAS, Rho, Rab, Ran, and Arf. ^21^^–^^23^ Primarily discovered as a viral component that initiated viral sarcoma in rats by Jennifer Harvey, ^24^^,^^25^ the oncogenic role of RAS has been known since then. Canonically, RAS superfamily proteins exist in either the active GTP-bound or the inactive GDP-bound state, their transformation being dependent on GTPase activating protein (GAP) and guanine nucleotide exchange factors (GEFs). ^26^^,^^27^

Until now, five isoforms of RAS proteins have been identified, namely HRAS, KRAS, NRAS, MRAS, and RRAS. ^8^ The HRAS, KRAS, and NRAS proteins share around 85% amino acid sequence identity and are widely expressed in cells. However, despite their similarities, studies have shown that KRAS is a fundamental protein in mouse development. ^28^ Upstream of RAS includes several signaling pathways like epidermal growth factor receptor (EGFR (ERBB1)), human epithelial growth factor receptor 2 (HER2 (ERBB2)), HER3 (ERBB3), and ERBB4, which mediates cellular proliferation and migratory actions. ^29^^,^^30^

RAS proteins require post-translational modification by farnesylation, adding a farnesyl isoprenoid moiety catalyzed by farnesyltransferase (FTase) to be biologically active. ^31^ This ensures the exact localization of RAS proteins at the inner surface of the plasma membrane, thus enabling them to recruit their target enzymes and initiate the signaling. ^32^^,^^33^ Upon activation, RAS induces numerous downstream proteins, such as Raf-1/mitogen-activated protein kinase pathway, phosphoinositide-3 kinase (PI3K), as well as the GEFs for the RAS-like (Ral) small GTPases (RalGEFs) and the Rac/Rho pathway. ^34^ Aberrant activation of RAS could lead to irregular cellular events such as cell proliferation, differentiation, and cancer. ^35^^,^^36^ Alteration of the RAS-MAPK pathway due to mutations in *RAS* or *RAF* genes has been very often reported. ^37^ RAS also activates BRAF, MEK1, and ERK, which regulate the transcription of genes that promote cancer. Moreover, RAS can activate the phosphatidylinositol 3-kinase (PI3K)-3-phosphoinositide-dependent protein kinase 1 (PDK1)-AKT pathway that facilitates cell growth and survival. RAS also activates the enzyme phospholipase C (PLC), that mediate calcium signaling and the protein kinase C (PKC). ^38^

### RAS signaling in cancer

RAS serves as a cell signaling protein downstream of various receptor tyrosine kinases and upstream of many signaling pathways associated with cancer. ^39^ When abnormally activated, RAS proteins initiate and collate many proliferative signaling pathways to exert a tumorigenic effect in tumor cells by significantly contributing to several aspects such as tumor growth, apoptosis, invasiveness, and angiogenesis. ^32^^,^^40^ Among the various cancer types, the global disease burden associated with *RAS* mutations accounts for approximately 19% of all cancer types engaged in tumorigenesis and tumor progression. ^40^^,^^41^ Single mutations at codon 12, 13 or 61 result in abnormal RAS functioning leading to hyperproliferative disorders such as cancer. ^42^

In humans, around 20% of all tumors show a gain-of-function mutation in one of the *RAS* genes. ^43^^,^^44^ Genomic sequencing analysis of human cancer specimens identified *KRAS* gene as the most frequently mutated gene, followed by *NRAS* and *HRAS.* ^45^^,^^46^ The incidence of *RAS* mutations in various cancers includes 57% in pancreatic cancer, 35% in the large intestine, 28% in the biliary tract, 17% in the small intestine, 16% in lung cancer, 15% in the endometrium, and 14% in OC. ^47^

An aberrant RAS signaling can be contributed by various mutations in closely related RAS proteins, importantly *KRAS* being most frequently mutated (about 85%), followed by *N RAS* (about 15%), and *HRAS* (less than 1%). ^9^^,^^41^^,^^48^ All these mutations are associated with GTPase activity of RAS, which prevents GAPs (GTPase Activating Proteins) from stimulating the hydrolysis of GTP on RAS, which in turn leads to the accumulation of RAS in the GTP-bound active form. ^9^ Moreover, mutations in the *KRAS* gene have been manifested to be involved in the pathogenesis of a variety of human tumors with pancreatic ductal adenocarcinoma (PDAC), colon cancer, and non-small cell lung cancer (NSCLC) showing the highest rate of *RAS* mutations. ^47^^,^^49^

A retrospective analysis by Zhu X. *et al*. reported a correlation between *RAS* mutational status and clinicopathological features among the colorectal cancer patients. Patients who foster mutant RAS has unique pathological characteristics, phenotypes, and staging. ^41^^,^^50^ Several studies have portrayed a remarkable correlation of *RAS* mutation with overall survival (OS) and poor prognosis. A comprehensive analysis conducted on metastatic colorectal cancer patients presented that, patients with a mutation in codon 12 of the *KRAS* gene demonstrated significantly poor OS compared to those with a wild-type mutation. However, the difference was insignificant for patients with *KRAS* mutation at codon 13. ^51^ Studies in pancreatic cancer cells highlight a novel approach to metabolic reprogramming created by combining glutamine inhibitors with chemotherapeutic drugs. This may be a potential therapeutic intervention to address the mutant *KRAS* that confers to chemoresistance in clinical studies. ^52^

Despite the enormous studies conducted, RAS, however, stood apart; it is allegedly termed “undruggable” and direct RAS inhibitor development proved exceedingly challenging. ^53^ Direct drugging of RAS protein was considered paradoxical due to the absence of a drug-binding pocket; consequently, studies started focusing on the proteins upstream and downstream of RAS that could help suppress the oncogenic signal. ^41^ Albeit drugging RAS had initial failures, tremendous efforts in understanding the complications of RAS have initiated new avenues for next-generation anti-RAS drug discovery by NCI (National Cancer Institute).

### RAS signaling in OC

OC, the uncontrolled division of malignant cells of ovaries, ^3^ is a leading gynecologic malignancy characterized by high mortality rates and poor prognostic outcomes. ^54^^,^^55^ In accordance with the American cancer society, in 2019, about 22,530 women were diagnosed with OC, and a mortality rate of 13,980 was reported. ^3^ Debulking surgery followed by chemotherapy and targeted therapy are the mainstay treatment strategies; however, most patients relapse due to chemoresistance. ^56^ There has been minimal progress in transitioning the remarkable strides in the multi-omics approach, including genomics, proteomics, and radiomics, into effective clinical administration of OC. ^3^ Despite the advancements in the treatment of OC, several studies report a relative five-year survival rate of less than 45%, and there has been no significant improvement in increasing the OS. Chemoresistance with the subsequent relapse and the side effects of the chemotherapeutic drugs urges the need to identify a better and reliable diagnostic, prognostic and predictive biomarker that would enable early detection and better screening. ^3^^,^^57^^,^^58^ Considering the heterogeneity, genetics, and molecular status of OC and the introduction of targeted therapies could significantly influence the management of OC. ^59^ The potential therapeutic targets identified for OC includes anti-VEGF/VEGFR angiogenic inhibitors, WNT inhibitors, non-VEGF angiogenic inhibitors, SONIC Hedgehog (SHH) inhibitors, NOTCH inhibitors, PARP inhibitors, EGFR inhibitors, folate receptor inhibitor, IGFR inhibitors, PI3K/PTEN inhibitors, and NF-kB inhibitor. ^60^^,^^61^

In a study involving 72 Japanese OC patients, *RAS* was found to be the third most commonly mutated gene with a frequency of 3.9% regardless of the histological subtypes, observed as mutually exclusive. Moreover, *KRAS* was more frequently found to be mutated in clear cell carcinoma patients (25.9%). ^62^

The *KRAS* mutations are the most commonly observed *RAS* isoforms, including *KRAS4A* and *KRAS4B,* wherein the mutations occur in exons 1 or 2. ^63^^,^^64^ Furthermore, the variant located in the 3′UTR of the *KRAS* gene (rs61764370 T > G), is associated with higher risks of several cancers such as OC. ^65^ However, it is noticed that *KRAS* mutations occur mostly in tissues with FIGO I and II than in FIGO III and IV stages, indicating *KRAS* mutation to be happening at an earlier part of cancer development. ^66^^,^^67^

Intriguingly, in OC, the most commonly mutated genes include *TP53, PIK3CA, ARID1A*, and *KRAS* disproportionately among the different histological subtypes with respect to their frequency of occurrence. ^68^^,^^69^ Moreover, *KRAS* mutation has been a common event in many histotypes of OC. ^70^^,^^71^

### RAS mutations in OC

Reports from previous studies confirm that the mutational status of *KRAS* shows an increasing trend from normal ovaries (0%) to benign mucinous ovarian tumors (BMOT) (57%), mucinous borderline ovarian tumors (MBOT) (90%), and mucinous OC (MOC) (76%) signifying its key involvement in the succession of benign tumors to aggressive OC. ^72^

In OC, *KRAS* mutations are observed in codons 12, 13, and 61, leading to a constitutively active RAS protein paving its way to an aberrant increase in tumor growth and malignant transformation. ^43^ *KRAS* mutation is also found to be a biomarker for poor outcomes and chemoresistance in OC. ^73^^,^^74^ In a comprehensive study, Mayr *et al*., assessed *KRAS* and *BRAF* mutations in a series of ovarian tumors and found that mutations usually occur at codons 12 and 13 of the *KRAS* gene with an occurrence rate of 3–11%. ^71^ Another study showed that *KRAS* mutations at codon 12 were more prevalent in borderline tumors than malignant ones. ^75^ Furthermore, a higher expression of Rab23, a member of RAS subfamily, is evidenced in OC tissues and is associated with the advanced FIGO stage. It is also known for its pivotal part in the malignant characteristic of OC and can be considered a potential therapeutic target for OC. ^76^

### OC subtypes

**Figure 1.**
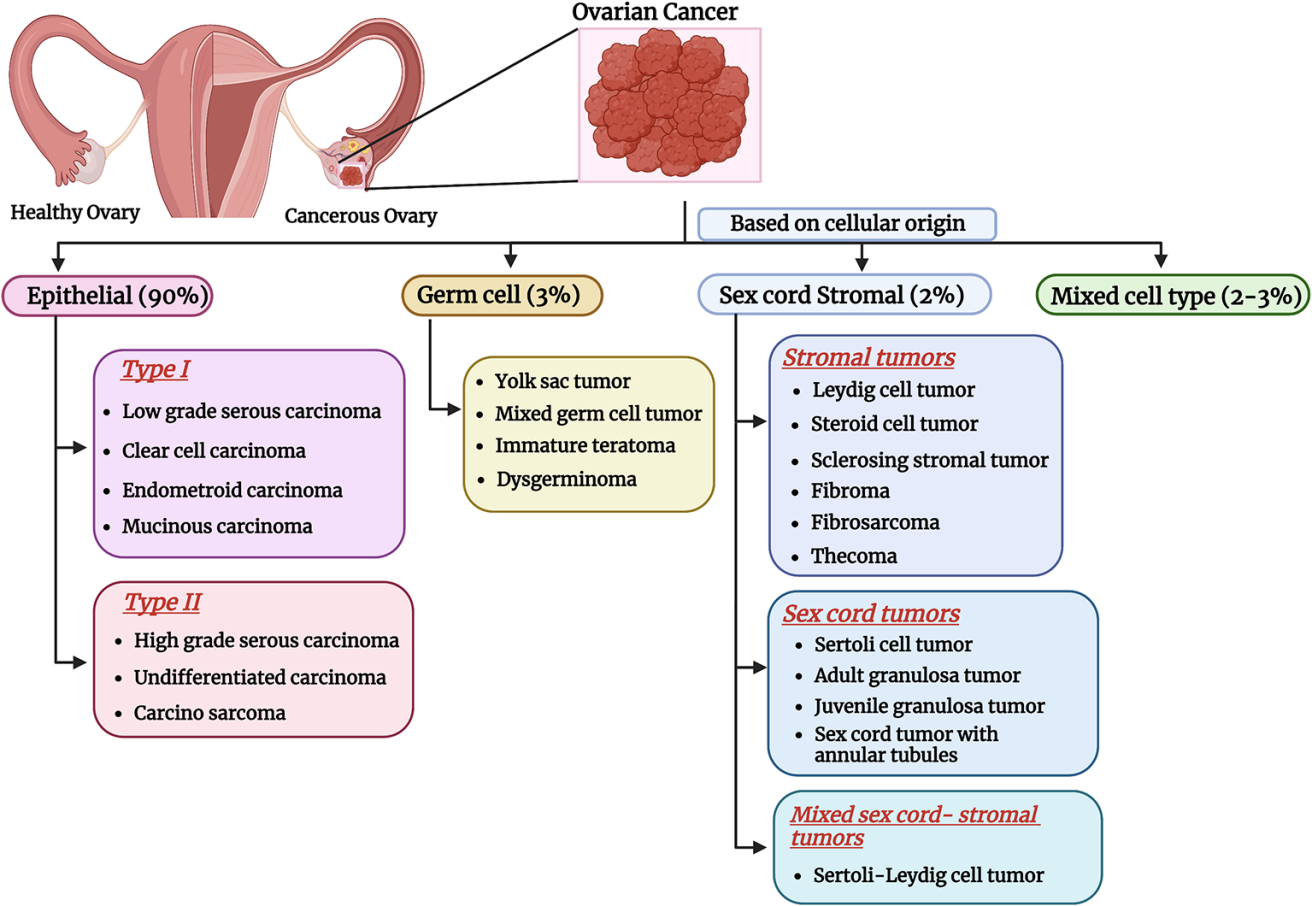
Classification of OC. OC is broadly classified into epithelial, germ cell, sex cord stromal and mixed cell types based on cellular origin, and subclassified based on the site of tumor occurrence and mutational status (created with biorender.com).

Genetically, OC represents a distinct subset of cancers with extensive genomic variations. ^77^ Broadly classified into epithelial OC (EOC), sex cord-stromal tumors (SCSTs), ovarian germ cell tumors (OGCTs), and small cell carcinoma of the ovary (SCCO), based on the origin of cancer, ^78^ EOC accounts for 90% of malignant ovarian neoplasms. ^79^ Currently, five major types of EOC is characterized: high-grade serous (HGSOC 70%), low-grade serous (LGSOC 10%), mucinous (MOC, 3%), endometrioid (EnOC, 10%), and clear-cell (OCCC,10%) carcinomas. ^77^^,^^80^^,^^81^ In addition, borderline ovarian tumors (BOT), also known as semi-malignant ovarian tumors, account for around 15% of EOC. ^82^ A broad classification of OC is represented in Figure 1.

In the next section, we briefly discuss about the frequency ( Table 1) of *RAS* mutations in different types of OC and their clinical relevance ( Table 2).

**Table 1.** Frequency of incidence of *KRAS* and *NRAS* mutations in different subtypes of OC.

OC subtype	*KRAS* mutations		*NRAS* mutations		Reference(s)
	Frequency	Mutational sites	Frequency	Mutational sites	
LGSOC	20–70%	A59T, G12A, G12C, G12D, G12F, G12R, G12S, G12V, G13D, G61H, Q61L	4–26.3%	G12C, G12V, G13C, G13V, Q61E, Q61H	^77^^,^^83^^–^^85^
HGSOC	5.9%		<1%		^5^^,^^86^^,^^87^
MOC	71%		-		^88^^,^^89^
EnOC	35%		-		^77^^,^^84^^,^^87^^,^^90^
OCCC	15%		-		^77^^,^^91^^–^^93^

**Table 2.** Mutational status and clinical relevance of *RAS* mutations in OC.

OC and subtypes	Specimen	Genes mutated	Pathways involved	Outcome	Ref.
OCCC	Patient specimens	*PIK3CA*	PIK3CA/AKT/mTOR pathway	PIK3CA could be a potential target	^94^
MOC	Patient specimens	*KRAS*	EGFR signaling	*KRAS* mutation at codon 12 and high titer of CA125 marker.	^95^
MOC	Frozen ovarian tumors	*KRAS* *BRAF* *NRAS*	MAPK pathway	Mutational status of mucinous carcinoma	^90^
EnOC	Human tissue specimen	*CTNNB1* *KRAS* *PTEN* *PIK3CA*	MAPK/RAS, WNT and PI3K pathways	TP53 and CTNNB1 can be potential prognostic markers	^96^
OCCC	Ovarian tumor tissue from patients	*ARID1A* *PIK3CA* *KRAS* *ERBB2* *ERBB3* *BRAF*	PI3/AKT and RTK/RAS pathways	PI3/AKT and RTK/RAS signaling pathways might be a prognostic marker	^97^
OCCC	Serum samples	*ARID1A* *PIK3CA* *KRAS*	PI3K/AKT, TP53, and ERBB2 pathways	Potential therapeutic target	^98^
OCCC	Patient sample	*PI3KCA* *KRAS*	PI3K/AKT pathway	Carcinogenesis and progression	^99^
SBOT	Patient sample	*KRAS* *BRAF*	RAS–RAF–MAP–MEK–ERK kinase pathway	Mutations associated with low-grade tumors	^71^^,^^100^
MBOT	Specimens from tumor bank	*KRAS* *TP53* *CDKN2A PIK3CA* *PTEN* *GNA11* *ERBB2*	RAS pathway	Different *RAS* mutation contributes to unique personality	^89^
MBOT	Ovarian tumors	*BRAF* *KRAS*	RAS-RAF-MEK-ERK signaling pathway MAPK pathway	Better prognostic biomarker in patients undergoing surgery	^101^
MOC	Ovarian tumors	*KRAS*	RAS-RAF-MEK-ERK signaling pathway MAPK pathway	borderline tumor progression to carcinomas	^101^
MOC	ovarian tissues	*BRAF HRAS KRAS* *MET* *NRAS PIK3CA*	RAS pathway	Better prognosis and low recurrence	^102^
MOC, EnOC, OCCC	Patient tumor samples	*KRAS*	Growth factor signaling DNA damage response p53 signaling Cell cycle control Apoptosis	Mutational status differs between distinct histological subtypes	^88^
MOC	Patient tissue sample	*KRAS*	RAS/Raf/MEK/ERK-pathway	Improved survival	^103^
LGSOC	Patient tumor tissue	*KRAS* *BRAF*	MAPK pathway	Better prognosis Improved OS	^104^
BOT with recurrent LGSOC	Patient tumor tissue	*KRAS G12V* *BRAF*	KRAS/RAF/MEK pathway	Shorter survival	^105^
MOC	Patient tumor specimen	*KRAS*	RAS pathway	Without *KRAS* mutation activation of RAS pathway could not be sustained	^106^
SBOT	Patient tumor specimen	*NRAS* *BRAF* *KRAS*	RAS pathway	NRAS may be an oncogenic driver	^107^
LGSOC	Patients tissue sample	*KRAS* *BRAF* *HRAS NRAS* *EIF1AX USP9X*	RAS/RAF/ERBB2-dependent cancer-associated pathways MAPK/ERBB2 signaling pathways	USP9X and EIF1AX novel driver of LGSOC	^83^
HGSOC	Patients tissue sample	*NRAS* *BRAF* *KRAS* *TP53*	RAS/RAF pathway	Co-occurrence of *TP53* mutation with mutations in RAS/RAF pathway	^108^
LGSOC	Patients tissue sample	*KRAS* *NRAS* *BRAF* *EIF1AX USP9X*	MAPK pathway	Low mutation rate of NRAS indicates a minor role in LGSOC development	^109^

### Low-Grade Serous OC (LGSOC)

LGSOC is a morphologically discrete subtype of OC, accounting for ~10% of serous carcinomas. ^110^ LGSOC is a distinct histological subtype that accounts for only 3% of EOC. It’s clinical characteristics include the diagnosis at a young age, prolonged OS, and chemoresistance. ^104^^,^^111^ In a previous study, up to 70% of LGSOCs were found to have *KRAS* mutation. ^105^ The LGSOCs have more frequent mutations in *KRAS, BRAF, ERBB2,* and *NRAS*, which are the signature genes involved in the MAPK signaling pathway. ^77^^,^^104^^,^^111^^,^^112^ LGSOC affects younger women aged between 43 and 47 years. ^113^ *KRAS* mutations are common (>70%) in recurrent LGSOC, ^105^ which usually occurs in SBOTs with LGSOC recurrence. In low-grade serous ovarian carcinoma, along with the *BRAF* and *KRAS* mutations, studies have also reported an *NRAS* mutation only in serous ovarian carcinoma, suggesting *NRAS* to be an oncogenic driver in serous OC. ^107^ Another study reported that *NRAS* mutations were present in 26.3% of LGSOC and were anticipated to be a potent initiator of tumorigenesis. ^83^ On the contrary, studies also suggest that the low mutation rates of *NRAS* alone may play only a minor role in the LGSOC development. ^109^ Somatic mutations in MAPK signaling pathway genes such as *KRAS, BRAF,* and *NRAS* are highly prevalent in LGSOC. ^110^ In the comprehensive genomic profiling study, Zhong *et al*. reported that *KRAS* mutation was a characteristic feature of LGSOC. ^108^ A report from Zuo *et al.* suggests that *KRAS* mutations are significantly associated with invasive implants of borderline serous tumor. They found that *KRAS* mutation is a significant prognostic indicator for tumor recurrence as higher recurrence rate of 71% was observed with patients carrying *KRAS* mutation wherein it was as low as 21% in patients without *KRAS* mutation. ^114^

In a study initiated by Xing *et al*., the mutational status of *NRAS* was determined at the hotspot region of exon 3 in 98 cases, and they detected *NRAS* Q61R mutations in 7.4% of LGSOC cases and 3.6% of non-invasive LGSOCs. This further suggests a lesser role of *NRAS* mutations in the occurrence of LGSOC. ^109^ These findings are also in accordance with previous studies where *NRAS* mutation was not observed in either SBT/APSTs or non-invasive LGSOCs. ^83^^,^^107^

Moreover, the co-existence of *NRAS* and *BRAF* mutations in LGSOC contradicts the type of mutations among MAPK pathway proteins. This further indicates that *NRAS* mutations might have a functional role in mediating other tumorigenic functions, such as invasion or tumor advancement ^115^^–^^117^; however, this warrants further investigation.

Chemoresistance is a challenging issue in the treatment of OC. ^118^ About 70% of the patients at the advanced stage are most refractory to platinum-based chemotherapy. ^68^^,^^119^ Previous reports suggest that LGSOCs are refractory to chemotherapy compared to the HGSOC. ^120^ The association of RAS with STAT3 has been proved to contribute to tumor growth, metastasis, and resistance to cisplatin treatment. This has also been known for the differential regulation of MAPK- and PI3K/AKT-mediated ERS and autophagy. ^118^ Moreover, platinum resistance was plausibly significant among the postmenopausal women with EOC among *KRAS* variant-positive patients than in the non- *KRAS* variant patients, making *KRAS* variant a prominent predictor of platinum resistance. Given the correlation between the *KRAS* variant and the resistance to platinum-based chemotherapy, the *KRAS* variant is considered as a biomarker of poor outcome. ^74^ Reports from Kato *et al*. showed that combination therapy using MEK inhibitor trametinib and aromatase inhibitor letrozole resulted in a better remarkable response in a woman with aggressive ER-positive, *KRAS*-mutated LGSOC. However, this effect was not observed when used as monotherapies. ^121^ Regardless of the enormous research, the chemoresistance due to *RAS* mutation still prevails as a major cause of concern and could be a promising approach to focus on the RAS initiated resistance to instigate a better treatment regimen for OC.

### High-grade serous OC (HGSOC)

The high-grade serous ovarian carcinoma (HGSOC) is the most common form of EOC, accounting for more than 70% of its frequency of occurrence and accounts for 70–80% of death in OC patients. ^122^^,^^123^ They portray a high degree of invasiveness and are mostly diagnosed at the later stage of development. They harbor some notable mutations that include: somatic *TP53* mutation, germline *BRCA1* and *BRCA2* defects, and lower frequencies of *RB1, PTEN,* and *NF1* mutations ^112^^,^^124^; scarcely they carry *KRAS* and *BRAF* mutations. ^5^

### Mucinous OC (MOC)

MOC, which is characterized by larger cells filled with fluid, is a rare subtype of EOC. ^125^ MOC are the histological subtype rarely reported in western countries and more commonly reported in Thailand. ^102^ The majority of the cases are presented as borderline tumors or at the early disease stage (FIGO I-II). ^77^^,^^89^ They have a better prognosis in case of early diagnosis but worse if diagnosed at the advanced stage. They have also been known for their poor response rates to platinum-based chemotherapy. ^126^ The most significant genetic alteration observed in mucinous carcinoma is the *KRAS* (71%) and *TP53* (57%) mutations. ^125^ Other mutations such as *PIK3CA* (8%) and *BRAF* (2%) have also been reported as an event of occurrence in MOC. ^102^ A higher amplification rate and overexpression of *ERBB2* and *ERBB3* mutation were also reported in mucinous ovarian tumors. ^90^ Mutations in codon 61 are rare in OC; moreover, it was found to frequently occur in mucinous adenocarcinomas and rarely in other common EOC. *KRAS* mutations are common in mucinous ovarian tumors and are identified in 40–50% of MOC cases. ^127^^–^^129^

In a study aimed at identifying the mutations in *KRAS* that were analyzed by direct genomic sequencing, the group determined that the overall frequency of *RAS* gene mutations was 27% found in most of the mucinous tumors. ^130^ The study portrayed about 11% of the cases with *KRAS* mutation at codon 12 and one with a mutation at codon 13 in ovarian tumors. They also demonstrated a noteworthy prognostic effect of *KRAS* mutation in EnOCs compared to the other histological subtypes. ^103^ A case study also reported the existence of the same *KRAS* mutation in the carcinoma cells and the functioning stromal cells, suggesting some regions possibly having a common origin. ^131^

Mackenzie *et al.* performed next-generation sequencing analysis with two MOC cases, previously established to have *ERBB2* over expression heterogeneity to identify sub-clonal populations containing either *KRAS* mutation or *ERBB2* amplification in order to establish if they were expressed independently or simultaneously. This study shows that *KRAS* mutations were the most frequently observed, with an incidence rate of 64.9% in MOC. However, concurrent ERBB2 amplification and *KRAS* mutation were observed in many cases. ^89^

Panyavaranant *et al.*'s report using 50 cases of primary mucinous ovarian carcinoma cases evaluated the relationship between genetic mutation and patients’ prognosis. Among the studied samples, 54% of the cases showed *KRAS* mutation; however, these cases had excellent prognoses. ^102^ A cohort study by Nodin *et al*. demonstrates an important correlation between *KRAS* mutations, mucinous histological subtype and progesterone receptor expression in OC patients. ^103^

### Endometrioid carcinoma (EnOC)

EnOC is associated with endometriosis and has a genetic resemblance to the endometrial tissue. ^112^ They account for about 10–20% of all OCs diagnosed at the early stage and are sensitive to platinum-based chemotherapy. ^77^ They are further classified as high- and low-grade endometrioid carcinoma, in which the high-grade closely resembles HGSOC clinically and molecularly. ^77^^,^^112^ The genes that are frequently mutated are *CTNNB1* ~50%, *PIK3CA* (phosphatidylinositol-4,5-bisphosphate 3-kinase catalytic subunit α) ~40%, *PTEN* ~25%, *KRAS* ~35%, and *ARID1A* (AT-rich interaction domain 1A) ~30%. Very few also harbor mutant *PPP2R1A.* ^77^^,^^111^^,^^112^^,^^132^ An elevated frequency of *KRAS* mutation in the human tissue specimens was hypothesized as the rationale for the chemoresistance and aggressiveness of EnOC. ^133^ A previous study also demonstrated the significant prevalence of the overexpression and amplification of *KRAS* gene in the aggressive phenotypes compared to the primary lesions. ^134^ Reports also suggest that the inflammatory, NF-κB, RAS, and TGF-β signaling pathways play a pivotal role in the pathogenesis of EnOC. ^135^ A retrospective analysis of the mouse model illustrates that the activation of the oncogenic *KRAS* allele provoked the epithelial component of the endometriosis to develop into benign epithelial lesions. They also reported that either the expression of the *KRAS* allele or conditional *PTEN* deletion in the ovarian epithelial surface resulted in preneoplastic ovarian lesions that showed an endometrioid glandular morphology. ^136^

### Ovarian clear cell carcinoma (OCCC)

Similar to endometrioid cancer, OCCC is also associated with the endometriosis and is most frequently observed in Asian countries, accounting for ~30% of cases in Japan and less than 10% of cases reported in Europe and the USA. ^111^^,^^112^ They are normally diagnosed at an earlier stage and are generally associated with resistance to platinum-based chemotherapy and poor prognosis. The most frequently observed mutations at the genomic level are *ARID1A* of ~50%, *PIK3CA* of ~50%, *KRAS* of ~14%, and *PTEN* at ~5%. ^77^^,^^91^^–^^93^ *KRAS* mutation in codon 12 exon 2 is observed in about 14% of OCCC, and an absence of *NRAS* and *BRAF* mutation. *KRAS* mutation was observed only in codon 12 and not in codon 13, validating the heterogeneity of EOC characterized by distinct molecular signatures. ^92^ Reports also suggest that, along with *KRAS*, the other gene components of MAPK pathway *PPP2R1A* and *ERBB2* were also frequently mutated in OCCCs and EnOCs. ^137^ The ovarian tumor tissue samples and their corresponding blood sample analysis from a group of Japanese women diagnosed with OCCC illustrated the alterations in genes involved in the RTK/RAS signaling cascade in 29% of cases. This includes the amplification of *ERBB2* (11%) and *ERBB3* (5%), and mutations of *ERBB2* (4%), *ERBB3* (7%), *KRAS* (9%), and *BRAF* (2%). ^97^ A whole genome sequencing of serum samples from the Korean patients diagnosed with OCCC revealed somatic mutation observed in genes that include *PIK3CA* (40%), *ARID1A* (40%), and *KRAS* (20%) in about 15 patients that correlates with *PI3K/AKT*, *TP53*, and *ERBB2* pathways. ^138^ In a retrospective analysis, *KRAS* mutations were detected among the Japanese patients in cells isolated from the regions of endometriosis adjacent to the site of carcinoma. Their DNA analysis of regions of endometriosis, atypical endometriosis and OCCC cells also displayed that *KRAS* mutation was observed only in the OCCC cells but not in endometriosis and atypical endometriosis. Their study hypothesized a correlation between *KRAS* mutation with malignant transformation of atypical endometriosis to OCCC. ^139^ A pyrosequencing analysis conducted on 63 patients diagnosed with OCCC revealed a higher prevalence of *PI3KCA* mutations of about 32% compared to the *KRAS* mutation, which existed at only about 13%. They also displayed a total absence of *BRAF* mutation and involvement of the PI3K/AKT pathway as an important event in carcinogenesis and progression, suggesting that OCCC harbor distinct molecular signatures with respect to other EnOC. ^140^

### Borderline ovarian tumor (BOT)

BOT are epithelial tumors characterized by variable nuclear atypia. ^141^ As first described by Taylor in 1929, this cancer was first described as a semi-malignant disease ^142^ characterized by a lack of stromal invasion. ^143^ Dobrzycka *et al*. analyzed the mutation at codon 12 of the *KRAS* gene in 78 women with ovarian tumors, including 64 invasive OCs and 14 BOTs, using an RFLP-PCR technique. *KRAS* codon 12 gene mutations were observed in 6.2% of OC tissue and 14.3% of BOTs. *KRAS* mutations were found to have a significantly higher frequency in MOC and BOT than serous tumors (p<0.01). They also found that mutation frequency was correlated with the histological type of tumor but not with stage, grade, or patient age. ^144^

Studies show that 88% of serous BOTs are presented with *KRAS* or *BRAF* mutations, suggesting their importance in developing SBOTs. ^71^^,^^100^ In mucinous BOT (MBOT), *KRAS* mutations are reported to be at a higher incidence level of 92.3%. ^89^ *RAS* mutation, along with *ERBB2* and *BRAF* mutations, can activate the MAPK pathway, ultimately leading to cell proliferation and cancer progression. ^145^ Ohnishi and his group have identified *KRAS* mutations in 43.8% of MOC cases. Specifically, the most predominant mutations were observed at *G12D* and *G13D.* In their study, the *KRAS*, *BRAF, TP53,* and *PIK3CA* mutational status in mucinous tumors of the ovary were identified using direct sequence analysis on 38 tumor specimens, including 16 MOCs, 10 MBOTs, and 12 MCAs. *KRAS* mutations were detected in MOC (43.8%) and MBOT (20%) cases and not in MCA cases. Moreover, the frequency of occurrence was higher in MBOT. These findings indicates that, *KRAS* mutations in MBOT might have a role in progression to MOC. ^101^

### Crosstalk between RAS and other signaling pathways in OC

RAS is found to crosstalk with many other tumor-inducing and tumor-suppressing pathways to regulate several physiological and pathological characteristics in OC. Mutant RAS interaction with p53, a tumor suppressor gene, is observed to regulate cisplatin resistance in OC via HDAC4- and HIF-1α-mediated regulation of apoptosis and autophagy. The group also found that ERK and AKT active *RAS* mutants are mutually suppressive, demonstrating that a crosstalk between RAS/p53 signaling and STAT3 regulates metastasis and chemoresistance in OC cells *via* the slug/MAPK and PI3K/AKT/mTOR- mediated regulation of epithelial to mesenchymal transition (EMT) and autophagy. ^118^ Downregulation of beclin 1, an important protein involved in autophagy, by RAS via PI3K/AKT and MEK/ERK pathway has been proved to inhibit autophagy. ^146^ Furthermore, loss of beclin1 activity is evidenced to be associated with several cancers including breast, ovarian and prostate cancer. ^147^

Isoprenyl cysteine carboxyl methyltransferase (Icmt), is an enzyme that catalyzes the final step of oncoproteins' prenylation, ^148^ and is known to have a role in growth and survival of various cancer cells. ^149^ Icmt expression is found to be upregulated in EOC patients irrespective of age and tumor stage. However, this upregulation is observed both at mRNA and protein levels. Moreover, OC cell lines with higher Icmt levels have been shown to express chemoresistance to drugs. Liu *et al.* showed RAS activation as a crucial effector for Icmt in OC cells. Using *in vitro* and *in vivo* studies, this group demonstrated that Icmt modulates RAS activation in OC cells and imparts chemoresistance in these cells. ^150^

FSH receptor binding inhibitor (FRBI) is an FSH antagonist that blocks FSH binding to its receptor. ^151^ FRBI is believed to suppress the tumorigenesis of OC by reducing cMyc, KRAS, and FSHR levels in the presence of FSH. Wei and his group reported that FRBI inhibited carcinogenesis and progression of OC by suppressing KRAS. ^152^

As reported earlier, RAS is activated by the son of sevenless (SOS1), whose expression is mediated by ligands that activate the aryl hydrocarbon receptor (AhR). This DRE-dependent activation of SOS is found to hasten cell proliferation in HepG2 hepatoma cells. ^153^ Though our group has already reported the involvement of AhR in inducing tumor proliferation in OC, the cross-talk between AhR and the RAS pathway still needs to be investigated thoroughly. ^154^ TCDD, an AhR activator, is found to induce RAS activity in hepatoma cells; however, studies contradict each other in terms of tissue specificity of this cross-talk. ^155^^–^^157^ Moreover, a microarray global expression analysis report has shown that RAS MAP kinase pathway activation observed in TCDD-treated human hepatoma cells to be AhR-dependent. ^158^

A recent study by Li *et al*. examined the effects of dysregulated micro-RNA expression in the progression of OC. The group tried to unveil the mechanism by which reduced expression of miR-324-3p could suppress OC proliferation. They found that WNK2, a cytoplasmic protein involved in ion transport, ^159^ is upregulated and promotes the growth and invasion of OC cells SKOV3 and CAOV3 by activating the RAS pathway. Moreover, phosphorylation modification levels of most proteins, most significantly RAS was observed when WNK2 was knocked down in SKOV3 and CAOV3 cell lines as analyzed by the Kyoto Encyclopedia of Genes and Genomes (KEGG) enrichment analysis. ^160^

**Figure 2.**
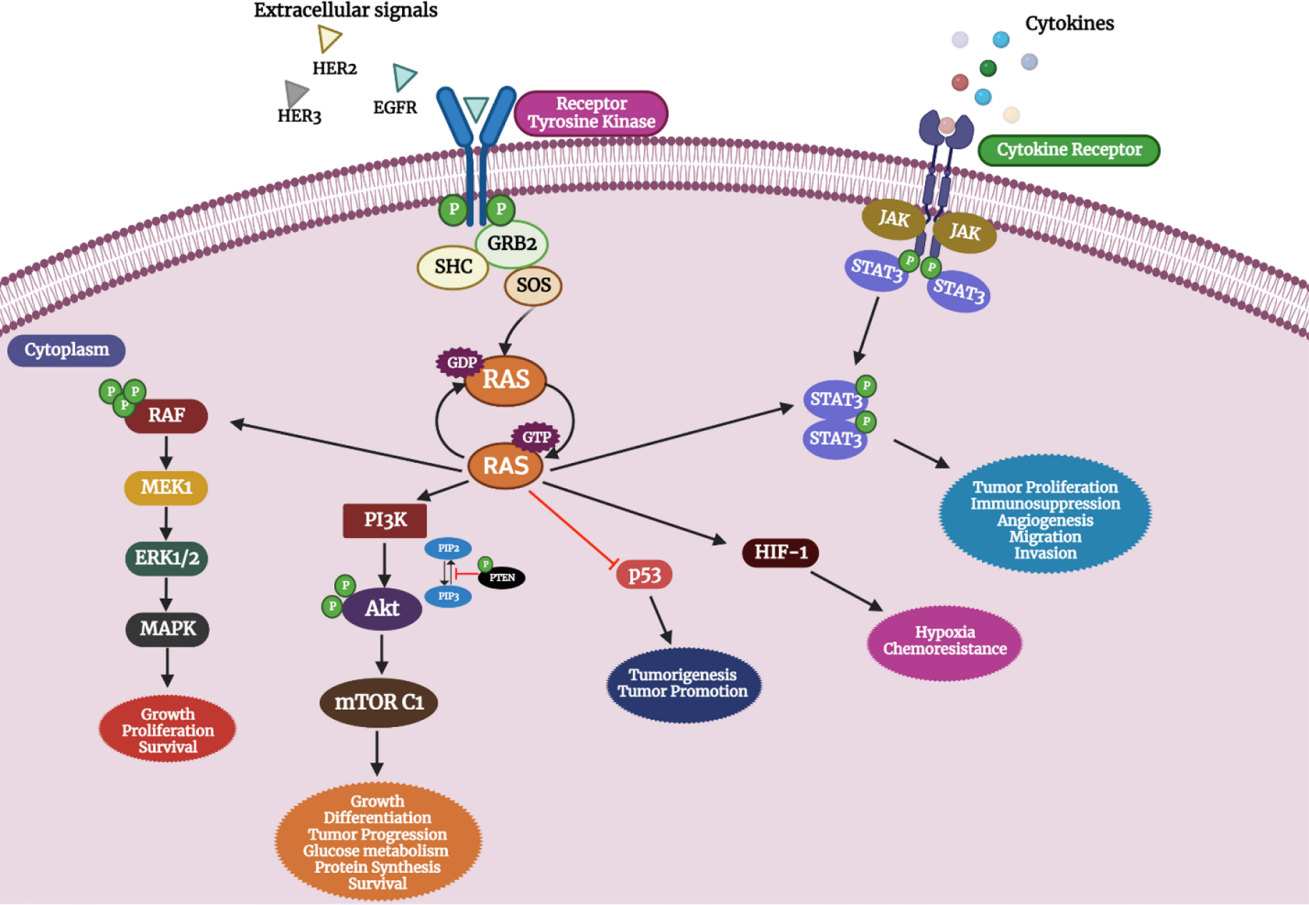
Effect of oncogenic activation of RAS signaling in OC. *RAS* mutation leads to aberrant activation of the RAS pathway leading to a cluster of other pathway activations involved in tumorigenesis (created with biorender.com).

Reports from a recent study reveal that tumor progression was abolished upon the inhibition of RAS GTPase-activating protein SH3 domain-binding protein 1 (G3BP1) ^161^ involved in the RAS signaling pathway which is also involved in the development of several cancers such as breast, colon, and gastric cancer. ^162^ Figure 2. depicts the effect of oncogenic activation of RAS in OC and its pathological outcome.

### Advancements in KRAS targeted therapy in OC

As a crucial gene mutated, the effects correlating with increased tumorigenesis, aggressiveness, and invasiveness in OC indicate RAS as a potential candidate for targeted therapy. RAS proteins are considered merely undruggable owing to their lack of drug binding pockets and to the very low binding affinity of GTP for RAS, which also makes GTP-competitive inhibitors inefficient. ^163^^–^^165^ Inhibiting RAS directly has proven challenging and has diverted researchers to consider alternate approaches targeting RAS downstream effectors. ^164^ Salirasib is a RAS inhibitor that interferes with the localization of RAS protein by removing the protein from the cellular membrane, resulting in reduced intracellular RAS, thereby affecting its downstream effectors. ^166^ Due to the extensive crosstalk of RAS with other pathways such as PI3K, the early attempt to inhibit a single pathway usually ended in promoting cellular resistance to chemotherapeutic drugs via a feedback loop. In the study conducted by Kim *et al*., when GDC, a pan inhibitor of PI3K, was administered to OC cells with *KRAS* mutation, the subsequent reduction in PI3K signaling resulted in over-expression of KRAS. However, when the inhibitor was combined with si-KRAS, this resulted in a synergistic anti-cancer effect in both ovarian OSE cell lines and allograft OC model impeding cell proliferation and migration and also inducing apoptosis in tumors *in vivo.* ^167^

Most studies published until now have confirmed that rather than targeting RAS itself, many targeted therapies use inhibitors of proteins involved in mutated RAS-activated downstream signaling pathways such as the RAF-MEK-ERK pathway. ^168^ Desai *et al*. evaluated the effect of Lifirafenib, primarily an RAF family kinase inhibitor, in tumors with KRAS mutations via dose escalation and observed antitumor activity in KRAS mutated endometrial cancer. ^169^

Even though profusely known to be undruggable, certain drugs that selectively target KRAS ^G12C^, not wild type or other KRAS mutants, have been discovered ^170^^,^^171^ AMG510, which potentially keeps RAS in an inactive GDP binding state, ^172^ MRTX8 _49_ (adagrasib), an oral selective inhibitor of RAS ^G12C^ that targets the mutant cysteine 12 of *KRAS*, ultimately keeping RAS in an inactive state, ^173^ and MRTX1133, a potent non-covalent inhibitor ^174^ are selective inhibitors of KRAS mutants, in which MRTX8 _49_ is currently in Phase I/II clinical studies. ^175^ Though these drugs are effective in attenuating RAS activity, ^176^^,^^177^ their effect on the cancer cells remains questionable due to some reports showing cell lines expressing KRAS ^G12C^, capable of sustaining the proliferating properties of cells despite the use of inhibitors, through adaptive feedback via wild-type RAS proteins. ^178^

Despite all the targeted therapy approaches defined, resistance to these inhibitors is developed. This includes mutations within the drug binding pockets, new KRASG12C protein production, feedback activations of the KRAS pathway, activation of both upstream and downstream mediators, *etc.* ^179^ *KRAS* mutation is a predictive marker of poor response to anti-EGFR monoclonal antibody therapies. ^180^^–^^183^

In a molecular profiling study with 55 patients with EOC, 35% were found to have ≥1 somatic mutation, including 23 *KRAS* and six *NRAS.* Out of this, 14 patients with *KRAS/NRAS* mutations treated with MEK inhibitor targeted combinations were subsequently enrolled in genotype-matched phase I or II trials. They observed that, in patients with *KRAS* mutation, a higher sensitivity to MEK inhibitors was observed, with seven patients showing a partial response, seven showing stable disease, and one showing disease progression. ^184^ The synthetic lethality therapeutic approach aims to inhibit both downstream pathway activation and feedback regulation of KRAS to ensure efficient therapy outcomes. One such drug is AZDD5483, a cyclin-dependent kinase effective on *KRAS* mutant tumor inhibition at G0/G1 phase, as confirmed in colorectal and pancreatic cancer. ^185^ In OC, this effect is achieved by combining MEK inhibitor (pimasertib) and PI3K/mTOR inhibitor (SAR245409, voxtalisib), identified by fluorescence resonance energy transfer imaging. ^186^

### OC stem cells associated with RAS functions involved in chemoresistance

**Figure 3.**
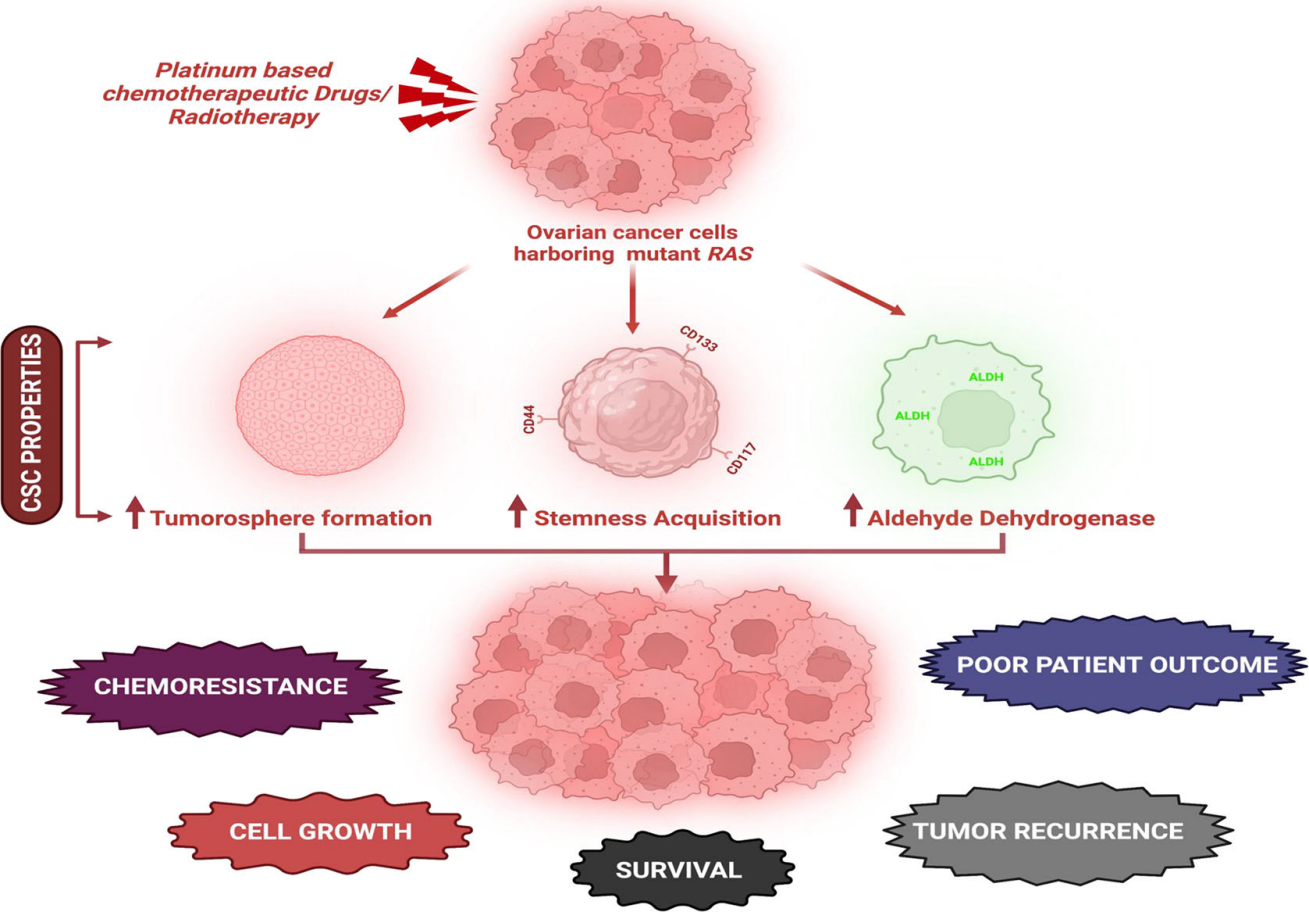
Mutant *RAS* associated acquisition of chemoresistance in OC. Active RAS mutants initiates CSC properties in OC resulting in chemoresistance, tumor recurrence and poor patient outcome (created with biorender.com).

Cancer stem cells (CSCs) are small subpopulation of cells within tumors with the potency for self-renewal, differentiation and tumorigenicity. ^187^ Accumulated pieces of evidence suggest a role of OC stem cells (OCSCs) in facilitating metastatic cascade, in frequent disease recurrence and increased resistance. ^188^^,^^189^ Few CSC markers, including ALDH1, CD44, CD117, and CD133 are considered to be useful predictive or prognostic biomarkers of OC. ^190^ The platinum-based chemotherapy resistance and tumor cell stemness is associated with the recurrence in HGSOC. In an aggressive murine model of OC, the stem phenotypes with a gain of *KRAS*, *MYC*, and *FAK* genes were found to be associated with intrinsic platinum resistance and tumorsphere formation. ^191^ Cisplatin-resistant EOC cell lines were found to significantly express OCSC markers and EMT activation triggered by activated PI3K/Akt/mTOR signaling indicating its correlation with chemoresistance in EOC. Moreover, treatment with an inhibitor BEZ235 in combination with cisplatin increased chemosensitivity in cisplatin-resistant EOC by inhibiting PI3K/Akt/mTOR signaling. ^192^ A gene expression analysis revealed OC patients with a significantly higher expression of *ROR1* having gene expression signatures associated with CSCs and shorter OS *. ROR1* was also involved in promoting tumor-cell growth, metastasis, and tumor initiation, making *ROR1* a potential target for therapies directed against OCSCs. ^193^ A recent analysis conducted by Zhang *et al*. to identify potential core signaling pathways of OCSCs using integrated transcriptome data of OCSCs isolated ALDH and side population, two distinctive stem cell surface markers. ^194^ A recent study by Shokouhifar *et al*. highlights the protocol for the generation of natural killer cells from umbilical cord blood hematopoietic stem cells by manipulating RAS/MAPK, IGF-1R and TGF-β signaling pathways that can be used for cancer immunotherapy. ^195^ RAS associated acquisition of chemoresistance in OC is depicted in Figure 3. Though the mechanism underlying chemoresistance in OC is still ambiguous, numerous such reports suggests the integral role of CSCs in chemoresistance and recurrence. Hence, OCSCs are a plausible therapeutic target in overcoming therapeutic resistance and recurrence.

## Conclusion

OC is a crucial disease characterized by chemoresistance, higher recurrence, and lower survival rates. A vast plethora of studies has already demonstrated the involvement and influence of several genes and their specific mutational statuses to be a major cause of OC, from the early development towards progression to invasion and metastasis. Studies confirm that *RAS* is one of the most mutated genes in OC, specifically, *KRAS* at codons 12,13 and 61. As a significant protein that has shown to be both downstream effector of several signaling pathways such as EGFR (ERBB1), HER2 (ERBB2), HER3 (ERBB3), and ERBB4, and upstream effector of RAF-1/MAPK, PI3K, RalGEFs, Rac/Rho, BRAF, MEK1, ERK, AKT, PLC and PKC, a mutation in *RAS* thereby causing hyperactivation of proteins could result in dysregulation ultimately leading to cancer initiation and proliferation. *KRAS* mutation, one of the majorly observed mutation in OC, is a predicted biomarker for poor clinical outcomes and chemoresistance. Involvement of genetic mutations, however, demanded targeted therapy initiation in OC in addition to the conservative therapeutic method of cytoreductive surgery followed by platinum-based chemotherapy. RAS was primarily believed to be undruggable due to the lack of drug binding pockets. Most publications confirm that targeting the downstream effectors of RAS paved more effect.

Moreover, as of its involvement in many other pathways such as cell proliferation, targeted therapy also had its disadvantages owing to the feedback loop, wherein inhibition of a single pathway ended up promoting chemoresistance. Recent advancements in targeting RAS utilize highly specific inhibitors that selectively target KRASG12C, not wild-type or other KRAS mutants. Targeting RAS, however, is much less explored in different histotypes of ovarian carcinoma and warrants further investigation.

## Data Availability

There are no data associated with this article.
