# Supplementary material for: Role of RAS signaling in ovarian cancer
Source: F1000Res. 2022 Nov 4;11:1253. [Version 1] doi: 10.12688/f1000research.126337.1 (PMC9669513; doi:10.12688/f1000research.126337.1)
Supplement: Peer review history

# Peer review: Reviewer response for version 1

**DOI:** 10.5256/f1000research.138739.r155264

**Published:** 2022-11-16

**Authors:** Haider Raza, Annie John

**Affiliations:** 1 Department of Biochemistry and Molecular Biology, College of Medicine and Health Sciences, United Arab Emirates University, Al Ain, United Arab Emirates; 2 United Arab Emirates University, Al Ain, United Arab Emirates

This is a comprehensive and interesting review on RAS family mutations in cancer focusing on ovarian cancer. In this review, the authors have described the role of RAS, which regulates several signaling pathways involved in cell proliferation, differentiation, and cell death/survival. The authors have focused on the mutational status of RAS, especially KRAS in mediating carcinogenesis and chemoresistance in ovarian cancer (OC).

 The authors have reviewed the subtypes of ovarian cancer, mutations in RAS, especially KRAS, the frequency of incidence, and the clinical relevance of the mutations on signaling pathways. They have also favorably tackled the problems involved in KRAS-targeted therapy and resistance in OC.

 However, a few minor issues need to be addressed:

1. The authors have schematically presented the classification of OC very well, based on cellular origin, with the various sub-types. Though the frequency of the other carcinomas is minimal compared to that of the epithelial, it would be a good idea to include at least some basic information regarding the tumors from other cellular origins.
2. In Table 2, the authors have given the mutational status and clinical relevance of RAS mutations. In the column, showing ‘Specimen’, the authors have given arbitrary descriptions like patient specimens or human tissue specimens etc. It would be good if the authors could identify and specify the specimen/tissues referred to in the given references.
3. The authors have mentioned the disadvantages of targeted therapy due to the feedback loop leading to chemoresistance. An elaboration on this would help the readers better understand the mechanism.
4. The discussion on the role of AhR and RAS activation is limited and referred to the author’s previous review which is not published yet (Ref #154?). An expansion on this subject (or access to the previous published review) may be beneficial to the readers owing to the wide range of AhR involvement in carcinogenesis, inflammation and hormonal signaling, including in OC.
5. A brief description on the pathophysiology and hormonal sensitivity of OC progression/regression, metastasis and chemoresistance/sensitivity would also improve the quality of the present review. Only cisplatin/platinum based-drug resistance has been discussed in this review. This can also be expanded by including other specific/precision-based therapies (e.g. in Figure 2 on radio-or other chemo-based therapies).

Is the review written in accessible language?

Yes

Are all factual statements correct and adequately supported by citations?

Yes

Are the conclusions drawn appropriate in the context of the current research literature?

Yes

Is the topic of the review discussed comprehensively in the context of the current literature?

Yes

Reviewer Expertise:

Biochemical and molecular bases of diseases, diabetes and cancer signalling. Molecular toxicology, oxidative stress, inflammation and mitochondrial dysfunction.

We confirm that we have read this submission and believe that we have an appropriate level of expertise to confirm that it is of an acceptable scientific standard.

# Peer review: Reviewer response for version 1

**DOI:** 10.5256/f1000research.138739.r155266

**Published:** 2022-11-14

**Authors:** Shams Tabrez

**Affiliations:** 1 Toxicology and Forensic Science Unit, King Fahd Medical Research Center, King Abdulaziz University, Jeddah, Saudi Arabia

In this article the authors have highlighted the role of the RAS family of proteins in the pathogenesis of ovarian cancers. The article has covered a wide range of functional aspects of RAS including mutational aspects of RAS and its association with various signaling pathways such as RAF-1/MAPK (mitogen-activated protein kinase), PI3K phosphoinositide-3 kinase (PI3K)/AKT, RalGEFs, Rac/Rho, BRAF (v-Raf murine sarcoma viral oncogene homolog B), MEK1 (mitogen-activated protein kinase kinase 1), ERK (extracellular signal-regulated kinase), PKB (protein kinase B), and PKC (protein kinase C) involved in cell proliferation as well as maintenance pathways thereby driving tumorigenesis and cancer cell propagation. 

 It is an important addition to the current state of literature and a very timely one too. The manuscript is well written, and literature extensively reviewed. There are just a few minor issues to take care of before possible indexing:

**Minor comments:**

1. It is will important to add a graphic abstract.
2. The full form of OC should be provided for use in the introduction section.
3. Editing/spelling checks in a few sections is required.

Is the review written in accessible language?

Yes

Are all factual statements correct and adequately supported by citations?

Yes

Are the conclusions drawn appropriate in the context of the current research literature?

Yes

Is the topic of the review discussed comprehensively in the context of the current literature?

Yes

Reviewer Expertise:

Cancer Biology; Molecular Signalling, Chemoprevention

I confirm that I have read this submission and believe that I have an appropriate level of expertise to confirm that it is of an acceptable scientific standard.
